# Online Assessment of Social Cognition in a Population of Gamers and Gamblers: Results of the eSMILE Study

**DOI:** 10.1007/s10899-023-10254-7

**Published:** 2023-09-24

**Authors:** Elodie Hurel, Marie Grall-Bronnec, Gaëlle Challet-Bouju

**Affiliations:** 1 CHU de Nantes, UIC Psychiatrie et Santé Mentale, Nantes Université, Nantes, France; 2 Univ Tours, CHU Nantes, INSERM, MethodS in Patient Centered Outcomes and HEalth ResEarch, SPHERE, Nantes Université, 44000 Nantes, France

**Keywords:** Gaming, Gambling, Facial emotion identification, Empathy, Social decision-making

## Abstract

The purpose of the eSMILE study was to explore social cognition (SC) in the two behavioural addictions (BAs) included in international classifications: gaming disorder and gambling disorder. In these disorders, cognitive functioning is involved in the development and maintenance of addiction. Nevertheless, SC have received less attention than other cognitive functions. The eSMILE study was conducted online and included 105 participants (gamers and gamblers). This study included: the Penn emotion recognition task, the Condensed and Revised Multifaced Empathy Test, the Interpersonal Reactivity Index, the Chicken Game, social metacognition questions, and the Toronto Alexithymia Scale. We analysed the relationships among SC measures, addiction levels, and behaviour frequency. For gamers, we showed that the higher their level of addiction was, the lower their self-confidence following the identification of basic emotions, although the more frequently they played, the better their performance on this task. Additionally, we found lower performance on the identification of more complex emotions by gamblers, which seems to be the result of their levels of addiction rather than the frequency of their gambling behaviour. This study contributes to our understanding of the cognitive processes underlying BAs. Additionally, working on SC abilities may be an additional management mode for BAs that could be added to existing treatments.

**Supplementary Information:**

The online version contains supplementary material available at 10.1007/s10899-023-10254-7.

## Introduction

### Behavioural Addictions (BAs)

Behavioural addictions (BAs) are nonsubstance addictions. Addiction is defined by the impossibility of controlling or stopping the behaviour and by repetition of the behaviour despite significant damage to several spheres of life (Goodman, 1990). This concept highlights a certain loss of control over a behaviour such as sport, food, sex, gaming, gaming or any behaviour that could fall into this category. Gambling disorder (GbD) was recognized as the first BA in 2013 in the fifth version of the Diagnostic and Statistical Manual of Mental Disorders (DSM-5) (American Psychiatry Association, 2013). The second BA included in international classifications of mental disorders was gaming disorder (GmD), which was included in the eleventh edition of the International Classification of Disease (ICD-11, (World Health Organization, 2018). Several theoretical models of addiction have been proposed to understand the development and maintenance of BAs. Among these models, the Interaction Person-Affect-Cognition-Emotion (I-PACE) model includes social anxiety and loneliness as predisposing factors that may influence the development of addiction. It also includes cognitive functioning as a moderator between reactions to addiction-related stimuli and decisions to behave in certain ways. In particular, the model postulates that inhibitory control over problematic behaviour is preserved in earlier stages of addiction but gradually decreases in later stages, especially when the person in question is confronted with addiction-related stimuli (Brand et al., 2019).

### Social Cognition (SC)

Cognitive functioning involves different forms of cognitive abilities, including social cognition (SC) abilities. SC is an umbrella term that covers all cognitive functions that permit an individual to perceive social information (such as the emotions displayed on faces) and thus interact and create relationships with others (Adolphs, 2001; Frith, 2008; Saxe, 2006). SC abilities include, for example, the capacity to identify facial emotions in others or abilities related to empathy (i.e., the competence to feel and understand the emotions of both others and oneself (Davis, 1980; Decety & Jackson, 2004). Social decision-making is also included in the category of SC abilities and refers to the capacity to make decisions affecting oneself and other persons that depend on the decisions of both parties (Rilling et al., 2008). Another SC capacity is social metacognition, which is the ability to assess one’s own abilities regarding a social object (Jost et al., 1998). Moreover, some authors have included alexithymia (i.e., the ability to identify and describe one’s own emotions (Sifneos et al., 1977) in the concept of SC (Etchepare & Prouteau, 2017). More specifically, previous research has shown that alexithymia can impact the ability to recognize facial emotions (Samur et al., 2013).

Among the various components of SC, two factors may modulate SC components: the depth of information processing (ranging from shallow to deep) and the nature of stimuli (i.e., cognitive—lacking an emotional context—or affective—with an emotional context) (Etchepare & Prouteau, 2017).

### BA and SC

Despite some common neural networks between addiction and SC components and the presence of alterations of SC that have been highlighted in the context of substance use disorders (Quednow, 2020), the literature concerning BA and cognitive assessment of SC remains scarce (Hurel et al., 2019). Regarding gambling, only two studies have been conducted, which showed an alteration of nonverbal emotional processing in faces and voices in individuals with GbD (Kornreich et al., 2016) and emphasized specificities in the attentional processing of facial cues by a group of nonproblematic poker players (Hurel et al., 2023a). Moreover, research using self-report questionnaires has also shown difficulties at the psychosocial level for pathological gamblers: alexithymia seems to be a significant vulnerability factor in the development of GbD (Gori et al., 2022), and there is an association between GbD and familial difficulties (Black et al., 2012; Cowlishaw et al., 2016). Additionally, analysis of self-reported mentalization abilities has indicated a link with GbD in adolescents (Ciccarelli et al., 2021) as well as with chasing behaviour (Nigro et al., 2019).

Research focusing on GmD and SC abilities using cognitive tasks has suggested an alteration of social stimuli perception (He et al., 2019; Jiang et al., 2020; Peng et al., 2017), a lower level of cooperation (Su et al., 2018), and a link between the symptoms of GmD and the ability to infer mental states (Aydın et al., 2020). Studies linking gaming and SC have been more numerous; these studies have not indicated any difficulties in empathy, but they have reported lower levels of cooperation for nonaddicted video game (VG) players (Jin & Li, 2017). Nevertheless, the multiplicity of methodologies and populations that have been assessed in the literature do not permit any generalization of these results (Hurel et al., 2023b). Additionally, self-reported level of loneliness seems to be a significant factor in the development of GmD (Paulus et al., 2018).

Thus, cognitive models, self-reports, and cognitive tasks all point in the same direction towards difficulties regarding SC abilities in the context of BA. However, more information regarding SC profiles in relation to BA could help clinicians adapt their treatments. At the scientific level, a better understanding of the cognitive features of BA can provide a better understanding of addictive processes, especially because BA offers a unique opportunity to explore the cognitive processes involved in addictions without biases resulting from the neurotoxic effects of psychoactive substances that have been found in substance use disorder studies.

In addition, studying SC abilities in gamers or gamblers, with or without addiction, and taking into account the frequency with which they engage in specific behaviours may also help us identify the role of SC in addictive processes and thereby enlighten models of addictive processes. Indeed, this approach would permit us to determine whether SC difficulties are linked to the repetition of the behaviour or are a core characteristic of addiction via an assessment of SC competencies in nonaddicted gamers/gamblers who exhibit frequent gaming/gambling behaviour.

### Objectives and Hypotheses

The eSMILE (onlinE aSsessMent of socIaL cognition of gamErs and gamblers) study aimed to characterize SC abilities in a population of gamers and gamblers while taking into account their frequency of gaming or gambling and their level of addiction. This study included both self-report questionnaires and cognitive tasks to better characterize the SC profile by collecting both subjective and objective data concerning several components of SC and obtaining a unified profile of each individual (Jeong et al., 2020). We assumed that only subjects with addictive behaviour would display SC alterations. Thus, we expected to observe a significant association between higher levels of addiction, lower SC competencies, and higher self-reported difficulties. Additionally, we did not expect a significant link between SC profile and frequency of behaviour. Indeed, we did not believe that the repetition of the behaviour engenders SC alterations but rather that SC difficulties are a core characteristic of addiction. We were also interested in exploring the role of alexithymia in SC among gamblers and gamers. We expected that, when including alexithymia levels in statistical models assessing the link between SC abilities and levels of addiction or behaviour frequency, the model including alexithymia would explain the results better than the model without alexithymia, i.e., including only levels of addiction or behaviour frequency. Indeed, high levels of alexithymia are found in GmD and GbD (Bonnaire et al., 2009; Mahapatra & Sharma, 2018), and alexithymia seems to impact the ability to process facial emotions (Grynberg et al., 2012). We wanted to control for this possible cofounding variable.

Finally, this study was designed during the COVID-19 pandemic, i.e., in a period in which it was not possible to assess volunteers for research in a face-to-face format in the lab. Thus, this study aimed to evaluate the feasibility of assessing SC profiles online. Beyond the adaptation to sanitary restrictions, online studies are supposed to have the advantage of gathering more participants than usual and allowing for the better generalization of results. This approach also permits participants to retain more time and flexibility (Reips & Krantz, 2010), and it increases ecological value by allowing them to use familiar materials (i.e., their computer) in a familiar environment (i.e., not in the lab) (Kim et al., 2019; Reips & Krantz, 2010). It also decreases bias resulting from the experimenter’s presence in the laboratory. Thus, this study included acceptability questions to assess whether participants found the instructions to be clear and whether they appreciated the online modality.

## Materials and Methods

### Participants

We planned to include four groups of 50 participants, two groups of gamers and two groups of gamblers, depending on their frequency of gaming or gambling. Participants were invited to participate by email and were recruited from a registry of volunteers for research that was created by our research team or via social networks from July 2021 to April 2022.

### Eligibility Criteria

To be included in the study, all participants were required to (1) be at least 18 years old and less than 65 years old and (2) have played their favourite game (gambling or VG) at least once during the 12 past months. The exclusion criteria were (1) difficulties reading or writing the French language, (2) having used a psychoactive substance in the past 12 h, (3) having a neurological history (such as epilepsy, severe brain injury, neurodegenerative disease), (4) having a diagnosis of a psychiatric or addictive disorder (with the exceptions of tobacco use disorder, gaming disorder and gambling disorder), (5) being pregnant or nursing, and (6) being under tutelage or curatorship.

To prevent individual participants from contributing to the study twice, we asked them whether they had already participated in the experiment before allowing them to access it.

The groups were created based on the frequency with which participants had engaged in their favourite behaviour during the past three months: high-frequency groups included participants who gambled or played a VG twice per week for at least 14 h, while low-frequency groups included participants who gambled or played a VG less than once per week. As a result, four groups were created: the low-frequency gambling group (LGB), the high-frequency gambling group (HGB), the low-frequency gaming group (LGM), and the high-frequency gaming group (HGM).

### Procedure

The website Psytoolkit (Stoet, 2010, 2017) was used to host the whole experiment. Participants were informed about the study and consented to participate online before being allowed access to the experiment. They also answered questions regarding the inclusion criteria and their frequency of gaming/gambling over the past three months to define their inclusion group.

We then collected certain information regarding sociodemographic characteristics and gambling/gaming (favourite type of gaming or gambling, duration of playing/gambling at the relevant frequency, number of hours played per month on average).

After answering these questions, participants were required to complete online cognitive tasks. Finally, they completed self-report questionnaires exploring their levels of addiction severity with respect to either gaming or gambling, alexithymia, empathy, depression and anxiety, and the acceptability of the online assessment. We also asked participants whether they had prior knowledge of the cognitive tasks and questionnaires used.

We chose to assess SC via cognitive tasks before the completion of questionnaires to avoid the effects of exhaustion and attentional decrease that may have occurred after answering all the questions.

### Sociodemographic and Gaming/Gambling Data

Sociodemographic data included age, sex, number of years of education, and living situation (alone, with family, with a partner). Gaming/gambling information included the choice of favourite game: (1) for the gambling groups, the participants could choose between poker, casino, lottery, horse race betting, sports betting, and other (free text field), while (2) for the gaming groups, the participants could choose between First-Person Shooter (FPS), Massively Multiplayer Online Role-Playing Game (MMORPG), Sandbox, Role Playing Game (RPG), Multiplayer Online Battle Arena (MOBA) and other (free text field). Participants were also requested to indicate the name of the game they played most frequently, the mean number of hours they played per month, and the durations for which they played at this frequency (in months).

### Clinical Assessment

#### Addiction Assessment

The Canadian Problem Gambling Index (CPGI, (Ferris & Wynne, 2001) was used to assess the addiction severity of gamblers (those who chose gambling as their favourite activity). This measure is a self-report questionnaire containing nine items regarding gambling habits during the past 12 months. Possible ratings include “never,“ “sometimes,“ “most of the time,“ and “nearly always”. Possible scores range from 0 to 27. This tool has good specificity (Ferris & Wynne, 2001).

The Ten-item Internet Gaming Disorder Test (IGTD10) (Király et al., 2019) was used to assess the addiction severity of gamers (those who chose gaming as their favourite activity). This tool contains ten items assessing nine criteria drawn from the DSM-5 (American Psychiatry Association, 2013) and the ICD-11 (World Health Organization, 2018) with respect to the past 12 months. Possible ratings include “never,“ “sometimes,“ and “often”. Possible scores range from 0 to 9. Research has indicated robust psychometric properties for this measure, which have been confirmed in several countries (King et al., 2020; Király et al., 2019).

Both questionnaires generated global scores that were used as continuous variables to indicate the level of addiction severity.

#### Level of Depression and Anxiety

The Hospital Anxiety and Depression Scale (HAD, (Zigmond & Snaith, 1983) was used to assess levels of depression and anxiety; this measure included 14 items, which were rated from 0 to 3.

### Social Cognition Assessment

#### Cognitive Tasks

Cognitive tasks appeared in the same order for all subjects, as indicated below.

##### Penn Emotion Recognition Task (ER-40) (Carter et al., 2009)

This task aimed to assess the ability to identify simple (basic) emotions. It included forty photographs of faces (20 women and 20 men; see (Gur et al., 2002) for details regarding pictures) with respect to which the participant was required to choose between sadness, fear, joy, anger, or neutrality (for photographs not displaying any emotion). For each emotion, four high-intensity emotion pictures (with emotions expressed at a high intensity) and 4 low-intensity emotion pictures (with emotion expressed at a lower intensity) were included. The variables recorded were reaction times and the number of errors. Four randomized lists were generated to pseudo-randomize the order of presentation among participants.

##### Condensed and Revised Multifaced Empathy Test (MET-CORE) (Grynberg et al., 2017)

This task aimed to assess the ability to identify complex emotions (by presenting pictures with a context; the emotions proposed included basic emotions and more complex emotions) and the degree of emotion sharing (a component of empathy). It included forty photographs (20 positive and 20 negative), each of which was viewed twice by the participant. In one of the presentations, the participant was required to choose the correct emotion displayed by the person in the photograph from a set of four choices. During the other presentation, participants rated the level of emotion displayed by the person in the photograph from 1 (none at all) to 9 (very strong). Regarding performance indicators, the software recorded the number of errors and reaction times for the identification questions and the rating and reaction times for the sharing component. A French team translated this task from German into French (Edele et al., 2013; Grynberg et al., 2017), which led to altered emotion identification abilities but preserved emotion sharing in a population of patients with substance use disorder (Grynberg et al., 2017). This task was used to complement the ER-40 because it permitted the assessment of facial emotion identification abilities in response to more complex stimuli and the subsequent assessment of this SC component at both levels (simple and complex facial emotion processing).

##### Social Decision-Making: The Chicken Game (CG) Paradigm

This task was an economic game assessing the level of cooperation. In each trial, participants were required to choose between two possible choices (i.e., a cooperative choice or a noncooperative choice). The outcome of the game depended on the participant’s own choice as well as on the choice of a partner in the game. In our study, the game partner was always the computer, but it was presented to the participant as either a computer (the “computer” condition) or another participant in the study (the “coplayer” condition). If both participants chose to cooperate, they both won ten points. If both participants defected, they each lost 30 points. If one cooperated and the other did not, the co-operator lost 10 points, and the noncooperator won 30 points. Matrices of payment were available to the participants throughout the task. Four trials were conducted: 2 for the “computer” condition and 2 for the “coplayer” condition (to preserve the credibility of a game played with another player, a waiting time of a few seconds was provided to simulate waiting for a connection from a coplayer). Choices (concerning whether to cooperate) made by the computer (either in the “computer” or the “coplayer” conditions) and the first condition to appear (“computer” or “coplayer”) were randomized. The software recorded choices and reaction times. In this task, the risk of defecting is more clearly identifiable than in the prisoner dilemma (another task that is often used to assess cooperation abilities). Not cooperating is the riskiest decision but also the one in which the gains are higher (de Heus et al., 2010). This task permitted to show diminished cooperation abilities in a population of participants with GmD (Su et al., 2018).

##### Social Metacognition

 We assessed social metacognition concurrently with the other cognitive tasks: once before the completion of the task but after the instructions were provided and again after completion of the task. On a scale from 0 to 100%, participants rated their performance on the task either prospectively (before the task) or retrospectively (after the task) (Nelson & Narens, 1994). This method did not add cognitive load to the completion of the task and was intended to assess appraisal and monitoring abilities (Quiles et al., 2014).

#### Self-reported Measures

##### Interpersonal Reactivity Index (IRI) (Davis, 1980)

This questionnaire assessed empathy abilities using four scales. The Personal Distress scale (PD) reflected the ability to feel shaken or moved by someone else’s distress. The Empathic Concern scale (EC) evaluated the ability to feel compassion or worry for someone. The Fantasy scale (FS) assessed the tendency to identify imaginatively with the thoughts and feelings of fictional characters. Finally, the Perspective Taking scale (PT) reflected the ability to adopt the perspective of someone else. The French version of this questionnaire was validated and demonstrated appropriate psychometrical properties (Gilet et al., 2013). In the present study, a seven-point scale with responses ranging from 1 (does not describe me well) to 7 (describes me very well) was used, as proposed in the French version of the questionnaire (Gilet et al., 2013).

##### Toronto Alexithymia Scale (TAS-20) (Loas et al., 1996)

This questionnaire assessed the level of alexithymia and included 20 questions that were answered on a scale ranging from 1 (complete disagreement) to 5 (complete agreement). It permitted us to calculate a global score and three subscores: difficulty identifying emotions (DIF), difficulty describing one’s own emotions (DDE), and thoughts aimed at the exterior instead of internal sensations (externally oriented thinking, EOT).

### Acceptability Assessment

Participants were asked to answer several questions on a scale ranging from 1 (totally agree) to 5 (totally disagree) regarding the acceptability of the study. Participants were required to indicate (1) the clearness of the instructions, (2) whether they would have visited the lab if the experiment had not been conducted online, and (3) whether they would have preferred to meet the experimenter.

### Statistical Analyses

Analyses were performed using R software (version 1.25001).

The original aim of the analyses was to compare performance on SC tasks and scores on self-report questionnaires between HGB and LGB on the one hand and between HGM and LGM on the other hand to assess the relationship between the frequency of the behaviour and SC profile. Subsequently, we intended to assess the link between addiction levels and SC performance. Nevertheless, incoherent data appeared with respect to the relation between the variable assessing the mean number of hours played on average per month and the variable in the inclusion criteria that asked about the mean frequency and duration of gaming or gambling during the last three months. As questions used for inclusion were binary questions (i.e., they were answered yes or no) and because participants did not know at that time whether they would continue participating in the study, thus indicating that their levels of attention and motivation were not high at this time, we considered this variable to be less relevant. We chose to use only the mean number of hours played per month on average as the most relevant variable for defining the frequency of the behaviour. This question was asked later, at a time when participants were more involved in the study in terms of motivation and attention. Moreover, participants were required to give a numeric answer, which requires more thinking and is thus supposed to be more reliable. Thus, we chose to keep only the analysis of the link between level of addiction and frequency of behaviour and SC measures.

In all cognitive tasks assessing reaction times (RTs), we excluded all RTs less than 250 ms because they were considered to be anticipative answers.

All correlations between variables assessing SC (self-reports and cognitive tasks) on the one hand and the mean number of hours played or addiction severity scores on the other hand were tested via Spearman correlations because these variables were not normally distributed (as determined by a Kruskal‒Wallis test). For all significant correlations, several independent linear regressions with performance on the corresponding SC task as the dependent variable and the mean number of hours played or addiction severity score as the independent variable were conducted. For each regression, two models were generated, one of which included only the significant correlation between SC measures, while the other added the alexithymia score level. Both models were compared by using a likelihood ratio test, the Akaike Information Criterion (AIC), and the Bayesian Information Criterion (BIC) to select the model that best fit our data. We used this strategy to explore whether the significant correlations observed were better explained by the level of addiction or the frequency of the behaviour.

Additionally, as our objective was to separate the effect of frequency on SC performance from that of addiction, we chose to assess the effects of both variables statistically for every variable that appeared to be correlated with level of addiction and frequency of behaviour.

When this situation appeared, we conducted a linear regression using the variable of interest as the dependent variable and the addiction severity score as the independent variable. We then added the mean number of hours played to a new model. Both models were compared by using a likelihood ratio test, the AIC, and the BIC to select the best model fitting our data.

Finally, participants’ metacognitive scores were normalized using the means and standard deviations scores of all answers. Correlations between these z scores and performance on SC tasks, behaviour frequency and addiction levels were subsequently estimated.

## Results

### Descriptive Analysis

#### Participant Descriptions

The final sample comprised 105 participants who completed the entire study; approximately half of these participants were gamers, while the other half were gamblers. Descriptive analyses of participants are provided in Table 1.

**Table 1:** Sociodemographic characteristics and gaming and gambling results

Variables	Gb	Gm
	Mean (sd) [min–max]	Mean (sd) [min–max]
Number of participants	56	49
Sociodemographic data		
Age	44.04 (12.30)	31.14 (10.46)
	[18–64]	[19–56]
Number of males	37 (66.07%)	30 (61.22%)
Number of years studying	14.07 (3.05)	15.63 (2.40)
	[7–23]	[12–23]
Living situation		
Alone	14 (25.00%)	13 (26.53%)
With family	24 (42.86%)	14 (28.57%)
With couple	14 (25.00%)	19 (38.78%)
Other (mainly colocation)	4 (7.14%)	3 (6.12%)
Gambling and gaming data		
Number of hours played per month	16.30 (37.39) 2 missing values	54.59 (62.51)
	[0–240]	[0–300]
Favourite game		
Poker	14 (25.00%)	
Casino	2 (3.57%)	
Lottery	21 (37.50%)	
Horse bets	7 (12.50%)	
Sport bets	9 (16.07%)	
Other	3 (5.36%)	
FPS		7 (14.29%)
MMORPG		5 (10.20%)
Sandbox		2 (4.08%)
RPG		16 (32.65%)
MOBA		8 (16.33%)
Other		11 (22.45%)

*Gb* gamblers, *Gm* gamers

#### Self-reports and Cognitive Tasks

Descriptive statistics of self-reports and cognitive tasks are available in supplementary material.

#### Acceptability Questions

Analysis of the acceptability questions are available in supplementary material. Participants found the instructions to be clear and did not prefer to meet researchers. There was no clear preference regarding whether they would visit the lab.

### Correlation Analysis

#### Correlations Between Self-reports and Gambling and Gaming Characteristics

Table 2 displays the correlations between answers on the self-report questionnaires and gambling or gaming characteristics. For both groups, we found a significant positive correlation between levels of depression and anxiety and the severity of addiction. That is, for all participants, a higher score of anxiety or depression was linked with a higher severity of addiction. Additionally, there were significant positive relationships between the addiction severity and the frequency of behaviour. That is, for all participants, the more frequently they played VGs or gambled, the more severe their addiction.

**Table 2:** Correlation coefficients for self-reports

	Mean number of hours played		Severity of addiction scores^a^	
	Gb	Gm	Gb	Gm
IRI—FS	− 0.05	0.21	− 0.01	0.24
IRI—PD	− 0.03	− 0.22	− 0.05	0.04
IRI—PT	− 0.04	− 0.03	0.02	0.01
IRI—EC	− 0.20	− 0.16	− 0.05	0.01
TAS—DIE	0.00	− 0.07	0.20	0.15
TAS—DDE	− 0.06	0.21	0.19	0.23
TAS—EOT	− 0.02	− 0.07	− 0.01	0.23
HAD—anxiety	0.01	− 0.27	0.32*	0.29*
HAD—depression	0.17	− 0.09	0.31*	0.31*
CPGI	0.55***			
IGTD10		0.40**		

p* = *p* < 0.05, p** = *p* < 0.01, p*** = *p* < 0.001, *CPGI* Canadian Problem Gambling Index, *Gb* gamblers, *Gm* gamers, *HAD* Hospital Anxiety and Depression scale, *IGTD10* Ten-item Internet Gaming Disorder Test, *IRI* Interpersonal Reactivity Index, *FS* Fantasy Scale, *PD* Personal Distress, *PT* Perspective Taking, *EC* Empathic Concern, *TAS-20* Toronto Alexithymia Scale, *DIE* Difficulty Identifying Emotions, *DDE* Difficulty Describing one’s own Emotions, *EOT* Externally Oriented Thinking

#### Correlations Between Cognitive Tasks and Gambling and Gaming Characteristics

Table 3 displays the results concerning the cognitive tasks in accordance with engagement in playing VGs or gambling games and the severity of addiction.

**Table 3:** Correlation coefficients for cognitive task

	Coefficient of correlation with mean number of hours played		Coefficient of correlation with scores on addiction questionnaires	
	Gb	Gm	Gb	Gm
*ER-40*				
Number of errors				
Total	− 0.12	− 0.04	0.10	− 0.15
Neutral faces	− 0.13	0.19	0.17	0.12
Angry faces	− 0.143	− 0.012	− 0.068	− 0.163
Fearful faces	0.21	− 0.06	0.11	0.01
Sad faces	0.00	− 0.34*	0.12	− 0.23
Joyful faces	0.05	0.01	0.16	− 0.05
Faces with low intensity emotion				
Neutral faces	− 0.01	− 0.17	− 0.01	− 0.11
Angry faces	− 0.16	0.09	− 0.10	− 0.06
Fearful faces	0.21	− 0.11	0.09	0.03
Sad faces	− 0.11	− 0.32*	− 0.08	− 0.06
Joyful faces	0.08	0.06	0.25	− 0.14
Faces with high intensity emotion				
Total	0.03	− 0.14	0.17	− 0.34*
Angry faces	− 0.04	− 0.11	0.01	− 0.18
Fearful faces	0.12	0.04	0.04	− 0.14
Sad faces	− 0.02	− 0.20	0.22	− 0.24
Joyful faces	− 0.01	0.05	− 0.03	0.06
MET-CORE				
Number of error total	0.23	0.28	0.40**	0.04
Number of errors for positive emotions	0.12	0.32*	0.25	0.07
Number of errors for negative emotions	0.28*	0.13	0.45***	0.02
Emotion sharing rating, global	0.12	0.01	0.11	− 0.04
Emotion sharing rating, positive	0.08	0.14	0.09	− 0.02
Emotion sharing rating, negative	0.12	− 0.05	0.14	− 0.04
CG				
Number of A choices, total	0.07	− 0.02	0.07	0.06
Number of A choices in the coplayer condition	0.03	0.03	0.10	0.03
Number of A choices in the computer condition	0.09	− 0.07	0.04	0.05

Bold value indicates p* = *p* < 0.05, p** = *p* < 0.01, p*** = *p* < 0.001

*CG* = Chicken Game, *Gb* gamblers, *Gm* gamers, *ER-40* Penn Emotion Recognition task, *MET-CORE* Condensed and Revised Multifaced Empathy Test, RT reaction times

Regarding the ER-40 task, a significant negative correlation was demonstrated for gamers between the frequency of playing VGs and the identification of sadness, such that a higher frequency of play was correlated with a lower number of errors on sad faces and sad faces with low intensity. That is, the more frequently gamers played, the better they were at identifying sadness, specifically on pictures with low emotion intensity. Additionally, a significant negative correlation was found among gamers between the severity of gaming disorder and the identification of high-intensity faces, such that a higher level of addiction severity was correlated with a lower number of errors on extreme faces, indicating that the higher the level of addiction severity, the better gamers’ ability to identify emotions on high-intensity faces.

In the MET-CORE, for gamers, there was a significant positive correlation between the frequency of behaviour and the number of errors for positive pictures. This correlation indicates that for gamers, the more hours they played, the more errors they made on positive pictures.

For gamblers, there was also a correlation between the frequency of behaviour and the number of errors on negative pictures; the more hours they played, the more errors they made on negative pictures. Additionally, the correlation between the level of addiction severity in gamblers and the number of errors on the MET-CORE was significant. That is, for gamblers, the more severe their addiction was, the more errors they made, both in total and specifically for negative pictures.

As both the frequency and level of severity were linked to the number of errors, we conducted a linear regression using the level of facial emotion identification abilities (number of errors) as the dependent variable and the addiction severity score as the independent variable. The model explaining errors and including only level of addiction demonstrated a better fit with the data than the model including both the frequency and the level of addiction. Additionally, for negative pictures (number of errors on negative pictures), the model including both frequency and addiction better explained our results than the model including only addiction, and both models provided better explanations than the model that included only the frequency of behaviour.

No link was found between gaming or gambling habits and empathy-sharing responses.

Finally, regarding the CG task, no analysis of correlation reached significance; see Table 3 for the specific numbers.

#### Metacognitive Scores and Correlations Between Performance on Tasks and Gambling and Gaming Characteristics

Normalized metacognitive scores (z scores) both before (pretask) and after (posttask) the cognitive tasks were linked with several variables; see Tables 4 and 5 for the results.

**Table 4:** Correlations between metacognitive scores and performance on cognitive tasks

Cognitive variables included in the correlation	Coefficient of correlation with the z score of the metacognitive score before the task		Coefficient of correlation with the z score of the metacognitive score after the task	
	Gb	Gm	Gb	Gm
*ER-40*				
Number of errors, total	− 0.02	0.14	− 0.12	− 0.12
Number of errors for faces with low intensity	0.04	− 0.02	0.16	− 0.04
Number of errors for faces with high intensity	0.02	0.22	0.03	− 0.16
*MET-CORE*				
Number of errors, total	− 0.23	0.15	0.09	− 0.18
Number of errors for positive pictures	− 0.28*	0.16	− 0.03	− 0.13
Number of errors for negative pictures	− 0.16	0.14	0.16	− 0.12
Rating, total	0.19	− 0.12	− 0.07	0.41*
Rating for positive pictures	− 0.01	− 0.01	− 0.01	0.26
Rating for negative pictures	0.23	− 0.19	− 0.07	0.40*
*CG*				
Number of cooperative choices	0.23	0.01	− 0.09	0.35*
Number of cooperative choices in the coplayer condition	0.44**	0.15	− 0.29*	0.19
Number of cooperative choices in the computer condition	− 0.05	− 0.14	0.16	0.30*

Bold value indicates p* = *p* < 0.05, p** = *p* < 0.01, p*** = *p* < 0.001

*CG* Chicken Game, *ER-40* Penn Emotion Recognition task, *Gb* gamblers, *Gm* gamers, *MET-CORE* Condensed and Revised Multifaced Empathy Test

**Table 5:** Correlations between metacognitive scores and gaming and gambling characteristics

	Number of hours played		Level of addiction	
	Gb	Gm	Gb	Gm
Metacognitive score before the ER-40	− 0.02	− 0.15	− 0.04	− 0.21
Metacognitive score after the ER-40	0.10	− 0.05	0.04	− 0.31*
Metacognitive score before the MET-CORE	− 0.25	− 0.14	− 0.18	0.08
Metacognitive score after the MER-CORE	− 0.14	0.01	0.05	− 0.08
Metacognitive score before the CG	0.18	0.13	0.27*	0.22
Metacognitive score after the CG	− 0.08	0.06	− 0.06	0.11

Bold value indicates p* = *p* < 0.05, p** = *p* < 0.01, p*** = *p* < 0.001

*CG* Chicken Game, *Gb* gamblers, *Gm* gamers, *ER-40* Penn Emotion Recognition task, *MET-CORE* Condensed and Revised Multifaced Empathy Test

First, there was a significant negative relationship between the number of errors on positive pictures made by gamblers in the context of the MET-CORE and their pretask metacognitive scores. Indeed, the more mistakes gamblers made, the less confident they were before the task.

There was a significant positive correlation for gamers between posttask metacognitive scores in the MET-CORE and ratings, specifically for negative pictures. This correlation indicates that, for gamers, the higher the level of emotion sharing rating with all pictures, specifically by negative pictures, the higher their level of confidence after the task.

Regarding the CG, for gamblers, there was a significant correlation between both pretask and posttask metacognitive scores and the number of cooperative choices with the other player. Before the task, this correlation was positive, indicating that the higher the participants’ levels of confidence before the task were, the more they cooperated with the “coplayer”. After the task, this correlation was negative. That is, the higher their levels of cooperation with the other person were, the lower their levels of confidence after the task.

In the analysis of correlations between the pretask or the posttask metacognitive z scores and the frequency of the behaviour, no correlation reached significance. For gamblers, there was a positive and significant correlation between the level of addiction severity and pretask metacognition in the CG. This relationship indicates that the higher the participant’s addiction severity was, the higher that participant’s level of confidence regarding his or her performance on the CG.

For gamers, a negative and significant correlation was found between levels of addiction severity and posttask metacognitive scores on the ER-40 task (r=-0.31, *p* = 0.04). This relationship indicates that for gamers, the more severe their addiction, the lower their confidence regarding their performance on the ER-40 task.

### Regression Analysis

All significant correlations were tested using a linear regression with or without alexithymia global score as a covariate, but only one model including alexithymia fit the data better than the simpler model (p value of models, LRT and AIC, and BIC are available in the supplementary material). The only SC component that was better explained by the model including the level of alexithymia was the model used to explain the posttask metacognitive score on the MET-CORE for gamers. Indeed, the model including the rated level of emotion sharing did not explain the data better than the model including both the rated level of emotion sharing and the level of alexithymia. That is, the metacognitive assessment of the ability to share an emotion is linked to the rating level as well as to the level of alexithymia.

## Discussion

The eSMILE study aimed to characterize SC abilities in accordance with gaming and gambling frequency using cognitive tasks and self-reports. We chose the online modality to (1) prevent people from moving during the pandemic and (2) assess the possibility of evaluating SC online. We included more than 100 participants in this study.

Regarding self-reports, the analysis showed that the more severe a person’s addiction was, the higher that person’s levels of anxiety and depression. Additionally, self-reported measures of empathy and alexithymia were not linked with the severity of addiction or the frequency of the behaviour.

Using cognitive tasks, we found a dissociation between simple and complex facial emotion identification abilities for gamers. For gamblers, this study found a negative link between the ability to identify a facial emotion and the level of addiction severity. Regarding social metacognition, particularities emerged for both groups regarding their assessments of facial identification abilities and levels of cooperation.

### Gamers’ Ability to Identify Facial Emotions and Metacognitive Abilities

As discussed above, we showed that the more frequently gamers played, the better they were at identifying sadness; the more severe their addiction was, the more accurate they were in identifying simple and highly intense facial emotions. These results are contrary to our expectations, even if research has shown that GmD subjects engage in a specific form of unconscious processing of emotional faces in the context of a population of patients with GmD (He et al., 2019; Peng et al., 2017). These studies did not assess the ability to correctly identify a facial emotion. Our results may also be linked with the fact that playing VGs may enhance cognitive functioning (Bediou et al., 2018). Indeed, playing VGs may improve the ability to efficiently process visual cues and may have thus helped our participants answer more accurately when identifying basic facial emotions as a result of this training.

Interestingly, we also showed that the more frequently gamers played, the lower their accuracy in identifying positive emotions in complex pictures. This finding may seem to contradict the results mentioned above. Nevertheless, those results were obtained in the context of a more complex task and may suggest that emotion processing is facilitated by playing VGs in the context of responding to simple stimuli but not complex stimuli. This dissociation is possible and has been demonstrated in individuals on the autism spectrum without an intellectual disability with respect to simple and complex prosody identification (Icht et al., 2021).

However, our study remains exploratory and does not permit us to make specific conclusions regarding the dissociation between simple and complex facial emotion identification. To answer this question, we would need to compare participants with a high frequency and a low frequency of gaming and control for addiction severity on a task such as the Reading the Mind in the Eyes Test. Indeed, this task comprises pictures that include only eyes, to which participants must attribute a complex emotional state. One study showed that the level of performance on this task with respect to negative emotions in among students might predict the severity of GmD (Aydın et al., 2020).

Contrary to our hypothesis, we did not find any link between the level of addiction and accuracy in identifying simple or complex facial emotions in gamers. This finding could be linked to our sample’s low level of addiction and the fact that they were not seeking treatment. Our sample did not represent patients in a clinical setting who faced with social difficulties. It would be interesting to use the ER-40 and the MET-CORE to investigate treatment-seeking patients to confirm the absence of any alteration in the ability to identify a facial emotion in the context of GmD while continuing to use self-reports to control for alexithymia because this ability impacts facial emotion processing (Grynberg et al., 2012).

Interestingly, the analysis of the metacognitive scores after the ER-40 task (assessing basic emotion recognition) showed that the higher gamers’ levels of addiction were, the lower their estimations of their performance. Our results suggest that pathological gamers tend to underestimate their ability to identify social stimuli. Metacognitive skills were proven to be deficient in a group of patients with cocaine use disorder (Balconi et al., 2014) and patients with Parkinson’s disease and GbD compared to patients with Parkinson’s disease but without GbD (Angioletti et al., 2020). In both studies, participants completed the Iowa Gambling Task (a decision-making task under conditions of uncertainty (Bechara, 2005)) and were required to estimate their performance on this task. These results suggest that addiction may be linked to a global deficit in metacognition. Indeed, our results showed that in cases of GmD, social metacognition might be altered. Nevertheless, further studies are needed to determine whether this tendency to underestimate one’s performance exists in the context of other tasks. If a deficit in metacognition appears in regard to several cognitive functions, this deficiency would be a common trait among cases of GbD, cocaine use disorder, and GmD. In this situation, metacognition deficits would be linked to addictive processes rather than the ingestion of psychoactive substances.

### Gambler’s Ability to Identify Facial Emotions

As discussed above, we showed that, for gamblers, the more frequently they played or the higher their level of addiction, the lower their accuracy in identifying complex negative emotions. These results seem to indicate that the errors made by gamblers may be linked to addiction rather than the frequency of the behaviour. Additionally, we controlled for alexithymia level and showed that these difficulties were not linked to this factor.

Using this framework, one study showed an alteration of emotion identification abilities in GbD patients with respect to both faces and voices (Kornreich et al., 2016). Thus, the research of Kornreich et al. and our study both suggest that SC, specifically the ability to identify emotions, is impaired in this context. Further studies comparing high frequency, low frequency, and GbD patients must be conducted to confirm this hypothesis. Indeed, we must investigate whether these difficulties may be linked more closely with a certain type of emotion (negative, positive ones) and whether other abilities are impaired. Actually, if facial emotion recognition is altered in cases of GbD, this situation could lead to other deficits because this type of information is used when inferring mental states (theory of mind, Achim et al., 2020; Premack & Woodruff, 1978). This type of difficulty would explain some of the difficulties that have reported by patients regarding familial difficulties, for example (Black et al., 2012).

Additionally, this component could be a target in therapies. Indeed, the use of cognitive remediation may help patients deal with social difficulties and enhance their social functioning (Kurtz et al., 2016; Kurtz & Richardson, 2012). This approach would lead to improving the social sphere, which is important in cases of addiction (Kim & Hodgins, 2018).

### Social Decision-Making

No significant relationships were found when assessing levels of cooperation in the social decision-making task. Some people indicated that they were familiar with the dilemma (even if they identified it as the prisoner dilemma instead of the CG). We decided to remove the scores of participants who were familiar with the prisoner’s dilemma (n = 4) to determine whether those participants may have biased our results. Nevertheless, the correlations among the number of cooperative choices, behaviour frequency, and levels of addiction remained nonsignificant.

Regarding the link with social metacognition, the higher gamblers’ levels of addiction severity were, the more confident they were before the CG. Nevertheless, level of addiction severity was not correlated with more cooperative choices. Additionally, the metacognitive scores of gamers tended to correlate with their behaviour towards the computer, while the metacognitive scores of gamblers tended to correlate with their levels of cooperation with the other player. A study of GmD subjects using the CG showed that participants with GmD tended to vary in terms of their level of cooperation depending on the partner with whom they were playing (Su et al., 2018). Even if we did not find that more cooperative choices were linked with addiction in one condition or the other, we can assume that gamers attributed their success to the fact that they cooperated with the computer, while gamblers anticipated that cooperation with the other player would be a good performance. Further research is needed to investigate the profiles of cooperation using different methods (more trials, different stories), but preserving both conditions seems important.

### Empathy Abilities in BA

No significant relationships were found between behaviour frequency or addiction and empathy measures using self-report and cognitive tasks. These results suggest the preservation of this ability, as has been found in the context of alcohol use disorder using the MET-CORE (Grynberg et al., 2017) and suggested by tasks completed by participants without GmD (Ferguson & Colwell, 2020; Gao et al., 2017; Kühn et al., 2018; Miedzobrodzka et al., 2021; Szycik et al., 2017). Nevertheless, lower empathic abilities have been reported with IRI in a GbD population (Tomei et al., 2017). Thus, further studies are needed to confirm the presence of empathy alterations in GbD by assessing a population of treatment-seeking patients. It is also necessary to assess the attribution component of empathy using another task, such as the Levels of Emotional Awareness Scale [LEAS (Lane et al., 1990)]. This scale demonstrated a lower level of emotional awareness in a population of substance use disorder patients (Carton et al., 2010). Using this task in the context of GbD and GmD patients may be a way of highlighting the dissociation between the BAs with respect to this SC component. This approach would permit the adaptation of treatment depending on the features of each BA.

### Difference Between Gamers and Gamblers

As showed in results above, this study did not highlight the same difficulties in gamers and gamblers. Indeed, gamblers’ ability to identify facial emotions may be affected while it seems to be the gamers’ ability to assess their own social abilities that seems altered. Despite the fact that in the CIM 11 (World Health Organization, 2018), criteria seem very close, characteristics of each practice may impact differently the cognition in general.

Indeed, gaming literature analysis showed the positive impact of gaming on cognitive function such as attention. Authors pointed out that difference only appear for the gamers that play the most, and that preferred game type may also impact attention and flexibility ability (Nuyens et al., 2019). Regarding social cognition, a systematic literature review showed that high frequency of gaming may be linked with improvement of moral competencies and changes in social decision making (Hurel et al., 2023b). Type of game could also impact this type of competencies because multiplayer games may need more theory of mind abilities. Motives to play could also impact the social investment of gamers and train (or not) social abilities.

For gambling, there is a continuum between luck and strategy. Indeed, gamble type vary the possibility to train certain cognitive functions. The most strategic and social gambling type may be poker (Palomäki et al., 2020). One study showed specificities in the allocation of attention of social cues in poker players compared to control (Hurel et al., 2023a).

Thus, it seems that both gaming and gambling may impact social cognition but for different reasons. Both practices may train certain social cognitive functions, depending on the type of game or gamble used. It may be interesting to compare social cognition performances between two groups of gamblers or gamers with different preferred game or gamble.

### The Online Experimentation

We wanted to assess the possibility of conducting such studies online. Our conclusions regarding this experiment are mixed. Indeed, our statistical plan was affected by instances in which inclusion criteria were not respected. This situation would not have happened if we had checked these criteria by phone, for example. Additionally, we encountered some difficulties regarding the number of inclusions. Indeed, while we planned to recruit 200 participants, we ultimately only recruited 105 over nine months. Throughout the duration of this study, we understood that it would have been easier to conduct this study via smartphone because many people do not have access to a computer, and diffusion via social media would have been facilitated. Moreover, including a reward for participation (such as a prize or gift certificate) could have increased participation and motivation.

However, the acceptability questions showed that participants might prefer not to meet the experimenter, thus suggesting that online participation may be easier. Our conclusion is that our methodology can be improved, specifically with respect to the construction of the task (accessibility on smartphones, prior confirmation of inclusion criterion).

## Conclusion

The eSMILE study showed that gamers and gamblers might present different difficulties with respect to SC. Indeed, gamers’ level of addiction can be linked with difficulties in assessing their own social abilities. GmD seems to be linked with difficulties identifying complex facial emotions. Our analyses found no effects of addiction or behaviour frequency on social decision-making and empathy. Assessing and improving SC abilities may be an alternative management mode for BAs that could be added to usual treatments, as in the case of one patient with sexual addiction (Hurel et al., 2021).

### Supplementary Information

Below is the link to the electronic supplementary material.

Supplementary file1 (DOCX 37 kb)

## Data Availability

The data that support the findings of this study are available from the corresponding author upon request.
