# Supplementary material for: Online Assessment of Social Cognition in a Population of Gamers and Gamblers: Results of the eSMILE Study
Source: J Gambl Stud. 2023 Sep 24;39(4):1611–33. doi: 10.1007/s10899-023-10254-7 (PMC10627996; doi:10.1007/s10899-023-10254-7)
Supplement: Supplementary file 1 — Supplementary file1 (DOCX 37 kb) [file 10899_2023_10254_MOESM1_ESM.docx]

Supplementary Table 1 Self-reported descriptive statistics

|  | Gb | Gm |
| --- | --- | --- |
|  | Mean (sd) | Mean (sd) |
|  | [min – max] | [min – max] |
| IRI | | |
| Fantasy scale | 30.45 (8.87) | 34.65 (7.98) |
|  | [15–49] | [20–48] |
| Personal distress scale | 24.38 (7.90) | 24.59 (8.18) |
|  | [9–43] | [7–44] |
| Perspective taking scale | 31.32 (6.592302) | 35.23 (6.61) |
|  | [15–42] | [22–49] |
| Empathic concern scale | 35.25 (6.81) | 36.98 (6.63) |
|  | [15–49] | [16–48] |
| TAS-20 | | |
| Difficulty identifying emotions | 17.04 (5.40) | 16.39 (5.13) |
|  | [7–35] | [7–28] |
| Difficulty describing emotions | 14.93 (4.98) | 13.35 (4.65) |
|  | [5–25] | [7–23] |
| Exterior operant thought | 19.21 (4.24) | 16.67 (4.43) |
|  | [8–28] | [10–27] |
| HAD | | |
| Anxiety | 7.68 (3.38) | 7.00 (3.83) |
|  | [3–19] | [1–16] |
| Depression | 4.79 (2.98) | 3.53 (2.69) |
|  | [0–12] | [0–13] |
| CPGI | 4.36 (5.59) |  |
|  | [0–27] |  |
| IGTD10 |  | 1.14 (1.52) |
|  |  | [0–7] |

CPGI=Canadian Problem Gambling Index; Gb=gamblers, Gm = gamers; HAD=Hospital Anxiety and Depression scale; IGTD10=Ten-item Internet Gaming Disorder Test; IRI=Interpersonal Reactivity Index; TAS-20=Toronto Alexithymia Scale

Supplementary Table 2 Descriptive statistics of cognitive tasks

|  | | Gbs | Gm | |
| --- | --- | --- | --- | --- |
|  | | Mean (sd) | Mean (sd) | |
|  | | [min – max] | [min – max] | |
| ER40 | | | | |
| Number of errors | Total | 6.41 (3.15) | 5.33 (2.13) | |
|  |  | [0–13] | [1–10] | |
|  | Neutral faces | 1.34 (1.95) | 0.71 (1.14) | |
|  |  | [0–8] | [0–5] | |
|  | Angry faces | 2.71 (1.46) | 2.14 (1.08) | |
|  |  | [0–7] | [0–4] | |
|  | Fearful faces | 0.91 (1.08) | 0.76 (1.11) | |
|  |  | [0–4] | [0–4] | |
|  | Sad faces | 1.29 (1.40) | 1.51 (1.14) | |
|  |  | [0–6] | [0–6] | |
|  | Joyful faces | 0.16 (0.37) | 0.20 (0.46) | |
|  |  | [0–1] | [0–2] | |
|  | Faces with low intensity | | | |
|  | Total | 3.73 (1.63) | 3.31 (1.37) | |
|  |  | [0–8] | [1–6] | |
|  | Angry faces | 2.29 (1.00) | 1.84 (0.99) | |
|  |  | [0–4] | [0–4] | |
|  | Fearful faces | 0.57 (0.78) | 0.43 (0.68) | |
|  |  | [0–3] | [0–2] | |
|  | Sad faces | 0.8 (0.82) | 0.98 (0.75) | |
|  |  | [0–3] | [0–3] | |
|  | Joyful faces | 0.07 (0.26) | 0.06 (0.24) | |
|  |  | [0–1] | [0–1] | |
|  | Faces with high intensity | | | |
|  | Total | 1.34 (1.55) | 1.31 (1.08) | |
|  |  | [0–6] | [0–4] | |
|  | Angry faces | 0.43 (0.71) | 0.31 (0.55) | |
|  |  | [0–3] | [0–2] | |
|  | Fearful faces | 0.34 (0.55) | 0.33 (0.59) | |
|  |  | [0–2] | [0–2] | |
|  | Sad faces | 0.48 (0.91) | 0.53 (0.71) | |
|  |  | [0–4] | [0–3] | |
|  | Joyful faces | 0.09 (0.29) | 0.14 (0.35) | |
|  |  | [0–1] | [0–1] | |
| MET-CORE | | | | |
| Number of errors identifying emotions | | 12.73 (3.61) | 12.08 (2.57) | |
|  |  | [5–20] | [6–18] | |
| Number of errors identifying positives emotions | | 6.96 (1.92) | 7.24 (1.51) | |
|  |  | [2–11] | [5–11] | |
| Number of errors identifying negative emotions | | 5.77 (2.30) | 4.84 (1.65) | |
|  |  | [1–11] | [1–8] | |
| Reaction times for emotion identification | | 4496.80 (1137.09) | 4755.30 (1174.29) | |
|  |  | [2842–7351] | [2442–8258] | |
| Emotion sharing rating of pictures | | 5.74 (1.45) | 5.48 (1.30) | |
|  |  | [2–9] | [2–8] | |
| Emotion sharing rating of positive pictures | | 6.37 (1.33) | 5.89 (1.39) | |
|  |  | [1–9] | [2–8] | |
| Emotion sharing rating of negative pictures | | 5.12 (1.99) | 5.06 (1.75) | |
|  |  | [1–9] | [1–8] | |
| CG (A is the cooperative choice) | | | | |
| Number of A choices | | 2.30 (1.26) | 2.65 (1.28) | |
| Number of A choices in the coplayer condition | | 1.27 (0.77) | 1.31 (0.80) | |
| Number of A choices in the computer condition | | 1.04 (0.83) | 1.35 (0.75) | |
| Metacognition | |  | | |
| Metacognition before the ER-40 (2 missing values) | | 75.98 (18.89) | | 78.04 (13.77) |
|  |  | [5–100] | | [50–100] |
| Metacognition after the ER-40 (2 missing values) | | 74.15 (13(44) | | 73.67 (11.11) |
|  | | [40–100] | | [50–95] |
| Metacognition before the MET-CORE (3 missing values) | | 69.33 (14.91) | | 73.60 (13.52) |
|  |  | [40–100] | | [50–100] |
| Metacognition after the MET-CORE | | 70.07 (15.27) | | 67.56 (13.44) |
|  | | [31–100] | | [40–90] |
| Metacognition before the CG | | 53.67 (17.37) | | 54.52 (18.60) |
|  | | [10–100] | | [10–100] |
| Metacognition after the CG | | 49.69 (28.06) | | 54.00 (32.42) |
|  | | [0–100] | | [0–100] |

CG=Chicken Game; ER-40 = Penn Emotion Recognition task; Gb = gamblers; Gm = gamers; MET-CORE = Condensed and Revised Multifaced Empathy Test

Supplementary Table 3 Descriptive statistics of acceptability questions

| Acceptability question | Mean (sd)  [min – max] |
| --- | --- |
| Instructions were clear. | 1.62 (0.92)  [1–5] |
| I would not have participated if I had to come to the lab. | 2.64 (1.56)  [1–5] |
| I would have preferred to meet the experimenter rather than answer online. | 3.19 (1.43)  [1–5] |

Ratings ranged from 1 (completely agree) to 5 (completely disagree)

Supplementary Table 4 Regression analysis

| **Dependant variables** | **Variables included in the model** | **p value of the model** | **p value of the comparison with the first model (LRT)** | **AIC** | **(BIC)** |
| --- | --- | --- | --- | --- | --- |
| **ER40** | | | | | |
| Number of errors on sad faces | Mean number of hours played for gamers | **0.042** |  | **152.431** | **158.106** |
|  | Mean number of hours played for gamers + TAS20 total score | 0.124 | 0.769 | 154.339 | 161.906 |
| Number of errors on sad faces with low intensity | Mean number of hours played for gamers | 0.103 |  | **113.013** | **118.689** |
|  | Mean number of hours played for gamers + TAS20 total score | 0.240 | 0.639 | 114.779 | 122.346 |
| Number of errors on extreme intensity faces | Score on the IGTD10 | **0.012** |  | **145.264** | **150.939** |
|  | Score on the IGTD10 + TAS20 total score | **0.020** | 0.205 | 145.581 | 153.148 |
| **MET-CORE** | | | | | |
| Number of errors | Score on the CPGI | **0.012** |  | **301.117** | **307.193** |
|  | Score on the CPGI + TAS20 total score | **0.032** | 0.412 | 302.412 | 310.513 |
|  | Score on the CPGI (without missing values from variables below) | **0.014** |  | **291.931** | **297.898** |
|  | Score on the CPGI (without missing values from variables in the model) + Mean number of hours played | **0.027** | 0.260 | 292.602 | 300.558 |
| Number of errors on positive pictures | Mean number of hours played for gamers | **0.026** |  | **179.032** | **184.708** |
|  | Mean number of hours played for gamers + TAS20 total score | **0.085** | 0.890 | 181.012 | 188.579 |
| Number of errors on negative pictures | Mean number of hours played for gamblers | 0.508 |  | 249.056 | 255.023 |
|  | Mean number of hours played + TAS20 total score | 0.805 | 0.992 | 251.056 | 259.012 |
|  | Score on the CPGI | **0.007** |  | **249.691** | **255.767** |
|  | Score on the CPGI + TAS20 total score | 0.021 | 0.497 | 251.206 | 259.307 |
|  | **Mean number of hours played** for gamblers **+ Score on the CPGI** | **0.012** | **0.002** | **242.093** | **250.049** |
| **Metacognition scores** | | | | | |
| *After the ER-40 task* | Score on the IGTD10 | 0.057 |  | **96.56** | **102.05** |
|  | Score on the IGTD10 + TAS20 total score | 0.120 | 0.42 | 97.86 | 105.17 |
| *Before the MET-CORE* | Number of errors on positive pictures | 0.257 |  | **76.49** | **81.98** |
|  | Number of errors on positive pictures + TAS20 | 0.474 | 0.64 | 78.25 | 85.57 |
| *After the MET-CORE* | Rating total for gamers | **0.005** |  | 90.27 | 95.76 |
|  | **Rating total for gamers + TAS20 total score** | **0.002** | **0.03** | **87.47** | **94.78** |
|  | Rating of negative pictures for gamers | **0.003** |  | 89.43 | **94.91** |
|  | Rating of negative pictures for gamers + tAS20 total score | **0.004** | 0.10 | **88.65** | 95.97 |
| *Before the CG task* | CPGI score | 0.447 |  | **127.46** | **133.37** |
|  | CPGI score + TAS20 total score | 0.751 | 0.97 | 129.46 | 137.34 |
|  | Number of cooperative choices with the other player for gamblers | **0.001** |  | **116.76** | **122.67** |
|  | Number of cooperative choices with the other player for gamblers + TAS20 total score | **0.005** | 0.80 | 118.69 | 126.58 |
| *After the CG task* | Number of cooperative choices with the other player for gamblers | 0.110 |  | **163.44** | **169.35** |
|  | Number of cooperative choices with the other player for gamblers + TAS20 total score | 0.231 | 0.52 | 165.01 | 172.89 |
|  | Number of cooperative choices for gamers | **0.022** |  | **150.98** | **156.47** |
|  | Number of cooperative choices for gamers + TAS20 total score | **0.069** | 0.67 | 152.79 | 160.11 |
|  | Number of cooperative choices with the computer for gamers | **0.047** |  | **152.33** | **157.82** |
|  | Number of cooperative choices with the computer for gamers + TAS20 | 0.108 | 0.45 | 153.73 | 161.04 |

**LRT = Likelihood Ratio Test; AIC = Akaike Information Criterion; BIC = Bayesian Information Criterion. Indicators of the better model are in bold (significant p value, minimum AIC and BIC).**
